# Life Satisfaction and Psychological Capital in Athletes with Physical Disabilities

**DOI:** 10.3390/bs13121010

**Published:** 2023-12-11

**Authors:** Olatz Zabala-Dominguez, Isabel Rubio Florido, Yolanda Lázaro Fernández, Erika Borrajo Mena

**Affiliations:** Deusto Sports and Society, Department of Physical Activity and Sports, Faculty of Education and Sport, University of Deusto, 48007 Bilbao, Spain; irubio@deusto.es (I.R.F.); yolanda.lazaro@deusto.es (Y.L.F.); borrajo.erika@deusto.es (E.B.M.)

**Keywords:** physical disability, well-being, sport psychological capital, psychological well-being, sport practice

## Abstract

Athletes with physical disabilities practice fewer sports than people without disabilities due to the difficulties and barriers they face in their daily lives. Sports are a powerful tool offering multiple physical, psychological and social benefits and act as a facilitator in their societal integration and inclusion. Today, more and more studies are analysing the psychological strengths of athletes, as well as their well-being. This research aimed to describe the levels of life satisfaction and psychological capital in a sample of physically disabled athletes according to the following variables: age, gender, degree of dependence, type of disability and level of competition. A structured questionnaire was distributed to 101 federated athletes with physical disabilities in the Basque Autonomous Community, with a valid return rate of 79 participants. A study of the relationship between variables was conducted using student’s *t*-test and ANOVA. A stepwise hierarchical multiple linear regression was also performed to assess the predictive capabilities of the analysed variables on life satisfaction. The findings showed that gender, degree of dependence and level of competition correlated with the psychological capital and life satisfaction of athletes with disabilities; male athletes scored higher in the self-efficacy and hope dimensions of psychological capital; athletes with lower levels of dependence scored higher on life satisfaction; and international athletes scored higher on psychological capital, specifically on the self-efficacy dimension. Finally, psychological capital and degree of dependence also had a significant influence on athletes’ life satisfaction.

## 1. Introduction

Physical disability is a motor impairment that limits daily activities to a greater or lesser extent, and people who suffer from it face daily obstacles and barriers that hinder their integration and inclusion in society, whether due to personal (generated by the disability itself) or environmental (caused by lack of accessibility) factors [1,2].

Sports are considered a powerful tool to achieve inclusion and barrier removal, as they offer multiple physical, psychological and social benefits [3]. These benefits would be even greater for people with disabilities, as they can improve their independence and health status, increase their self-confidence, improve their mood, reduce stress and anxiety and favour social relationships [4,5]. Despite these multiple benefits, studies have shown that people with disabilities do not meet the minimum physical activity recommendations proposed by the World Health Organization (WHO) and, consequently, have a higher risk of suffering from health diseases related to sedentary lifestyles [6,7].

Sports in this population are called ‘sports for people with disabilities’ and may refer to those that are adapted to the specific needs of each disability [8]. These adaptations facilitate the practice of conventional sports by modifying their structures, rules and materials as well as human resources [9]. However, adaptations may sometimes be unnecessary because some sports have already been designed based on the specific needs of the group, as in the case of boccia or slalom [10]. In any case, an increasing number of studies have analysed the physical and psychological benefits of sports for athletes with disabilities [11,12]. These benefits include increased energy expenditure as well as improved lung capacity, muscle tone, self-esteem, self-efficacy and well-being [10,13].

Well-being can be analysed from two perspectives: hedonic and eudaimonic. On the one hand, hedonic or subjective well-being consists of affective-emotional and cognitive aspects and is used to evaluate the level of an individual’s life satisfaction [14]. Therefore, life satisfaction is considered the result of a person’s evaluation of his or her life trajectory, in general, or of specific aspects, such as family, friends, and so on [15]. A high degree of life satisfaction is related to better mental health and decreased levels of depression [14,16,17].

On the other hand, eudaimonic or psychological well-being is associated with functional capacity. It includes values such as the feeling of being alive or those related to personal growth [18,19]. In relation to this type of well-being, a positive affective state and a high psychological capacity facilitate optimal performance among athletes [12,20,21,22]. Along these lines, the latest studies on the capacities and strengths of athletes have included the assessment of psychological capital, a novel tool that comprehensively analyses the psychological strengths of subjects, thus offering results in the field of well-being and athletic performance [23,24]. To assess these capabilities, psychological capital is identified according to four factors: self-efficacy, optimism, hope and resilience. Luthans et al. [25] define them as follows: ‘(1) having confidence (self-efficacy) to make the necessary efforts in order to achieve success in challenging tasks; (2) making a positive attribution (optimism) about being successful now and in a future; (3) preserving in the achievement of goals, and when necessary, redirecting the path toward goals (hope) to succeed; [and] (4) when beset by problems and adversities, sustaining and recovering and even overcoming them (resilience) to achieve success’ (p. 2).

Positive psychological capital, such as psychological well-being, has its origin in positive psychology, and its study has traditionally focused on the organisational sphere, with the aim of improving the productivity and well-being of workers [23]. Despite this, an increasing number of studies have measured psychological capital in sports because of its powerful results [12,24,26]. In addition, several studies have analysed psychological capital together with life satisfaction, obtaining a positive relationship between the two variables [15,27,28,29].

In studying the subjective well-being and psychological capital of athletes with physical disabilities, it is essential to consider the influence of personal and sporting factors. Among the most relevant factors are age, sex, degree of dependence, origin of disability and level of sports competition. The literature has shown no differences among these sociodemographic variables. For example, regarding age and sex, Hernández et al. [3] analysed the psychological characteristics of athletes with physical disabilities and reported that there were no differences according to age and sex. Tejero-González et al. [30] analysed life satisfaction and self-efficacy and did not find a relationship between age and sex. Similarly, another study on athletes with disabilities did not find any relationship between age and psycho-psychological strengths and subjective vitality [2].

With respect to gender, a significant disparity has been found between the participation of men and women. According to the data, the rate of physical disability in the population affected women to a greater extent [31]; however, in scientific research, the rate of female participation was generally low. For example, in the study by Di Cango et al. [32] and Dursun et al. [33], no female athletes participated and in the study by Tejero-Gonzalez et al. [31], they represented only 4.8% of the total. This lack of participation hinders the representativeness of the results.

Regarding the characteristics of the disability, studies have shown that athletes with a lower degree of dependence tend to have a higher level of life satisfaction [34,35,36]. Regarding the type of disability, athletes with acquired disabilities generally have a lower level of life satisfaction [37]. In terms of psychological strengths, several studies have shown that athletes with acquired disabilities have the highest scores [36,38]. Mira et al. [39] recently showed that people with acquired disabilities have higher levels of subjective and psychological well-being.

Finally, athletes with increased levels of competition have higher values of psychological strengths, scoring higher in the areas of self-efficacy, perceived competence and resilience [40,41]. Regarding life satisfaction, despite not finding significant results, Tejero-González et al. [30] and other researchers have shown that athletes with physical disabilities who engage in high-level competitions tend to have higher perceptions of life satisfaction [42,43].

The objective of the current research was to describe the levels of life satisfaction and psychological capital in a sample of physically disabled athletes according to several variables, namely, age, gender, degree of dependence, type of disability and level of competition.

Drawing from these previous studies, we propose the following research hypotheses:

**Hypothesis 1** **(H1):** *Older athletes have higher PsyCap scores than younger athletes.*

**Hypothesis 2** **(H2):** *Male athletes have a higher PsyCap scores than female athletes.*

**Hypothesis 3** **(H3):** *Athletes with a lower degree of dependence have higher values of life satisfaction.*

**Hypothesis 4** **(H4):** *Athletes with an innate disability have a higher life satisfaction than athletes with an acquired disability.*

**Hypothesis 5** **(H5):** *Athletes competing at international level have a higher PsyCap than athletes competing at regional or national level.*

## 2. Materials and Methods

### 2.1. Participants

A total of 79 athletes with physical disabilities from the Basque Autonomous Community voluntarily participated out of 101 athletes contacted through federations, clubs and online resources. In the data collection process 22 athletes did not respond to the questionnaire. Their ages ranged from 18 to 68 years (M = 39.94; SD = 12.85). In terms of sex, 86.1% were men and 13.9% were women. Regarding the characteristics of disability, 36.7% had an innate disability, while 63.3% had an acquired disability. About 36.7% had a medial injury. A total of 36.7% had a spinal cord injury, 21.5% had an amputation, 11.4% had cerebral palsy and 30.4% reported other types of disability, including muscular dystrophy, poliomyelitis and spastic paraparesis. Regarding the degree of dependence, 45.6% had a degree of dependence 0 (i.e., no recognised degree), 26.6% had a degree of dependence I (moderate) and 27.8% had degrees of dependence II and III (severe/highly severe). Regarding the athletes’ level of competition, 39.2%, 31.6% and 29.1% competed at the international, national and regional levels, respectively.

Analysing the personal characteristics of the sample, we observed that most of the athletes competing at the international and regional levels were men (N = 28) and women (N = 7), respectively. With regard to age, athletes with the highest average (M = 41.74) participated in competitions at the regional level compared with those who competed at the national (M = 39.12) or international (M = 39.26) levels. Regarding the origin of the disability, the majority of athletes with acquired disabilities competed at the international level (N = 23) compared with athletes with innate disabilities (N = 8). The characteristics are shown in Table 1.

### 2.2. Measurements

#### 2.2.1. Demographics

The questionnaire consisted of questions regarding age, gender, educational level, employment status, origin of disability (innate/acquired), type of injury (spinal cord/amputation/cerebral palsy/other), degree of dependency (Grade 0: ≤33%/Grade I: 33–64%/Grade II: 65–74% and III ≥ 75%) and level of competition (international/national/regional). The degree of dependency follows the values used by the disability assessment system in the Basque Country: Grade 0: from 0–24 points; Grade I: from 25 to 49 points; Grade II: from 50–74 points and Grade III: from 75 to 100 points [44].

#### 2.2.2. Life Satisfaction Scale

The Satisfaction with Life Scale (SWLS) [45], adapted and validated for the Spanish context [46], was used to measure life satisfaction. The scale consists of five items with seven response options (1 = ‘not at all agree’ and 7 = ‘strongly agree’). The items are as follows: ‘In general, my life corresponds to my ideals’, ‘My living conditions are very good, ‘I am satisfied with my life’, ‘So far, I have achieved important things in life’, and ‘If I were born again, I would wish to have the same life’. In the present study, the scale showed an internal consistency or Cronbach’s alpha reliability coefficient of 0.84.

#### 2.2.3. Sports Capital Scale (Psycap)

Sport psychological capital was measured with the Sport Psychological Capital Questionnaire (SPCQ) by Borrajo et al. [24]. This scale consists of 18 items and includes four dimensions: Efficacy (six items, e.g., ‘I feel confident analysing a long-term problem to find a solution’), Hope (six items, e.g., ‘I can think of many ways to achieve my current sport goals’), Resilience (four items, e.g., ‘I usually handle difficulties one way and another in sport practice’) and Optimism (two items, e.g., ‘When things are uncertain for me in sports practice, I usually hope for the best’). The participating athletes were asked to indicate on what average they agreed with the 18 statements on a 6-point Likert-type scale, with 1 = ‘strongly disagree’ to 6 = ‘strongly agree’. In the present study, the reliability analysis yielded a value of alpha = 0.89.

### 2.3. Procedure

This study used a quantitative approach through descriptive analysis and a correlational design in order to examine and understand the relationships between variables. Data collection was conducted through a survey, wherein participants answered data on sociodemographic variables, life satisfaction and sport psychological capital. The online questionnaire can be completed at an approximate duration of 10 min and was sent by e-mail to the Basque Adapted Sport Federation. The survey was conducted through the Qualtrics platform. The federation proceeded to disseminate it among sports clubs throughout the Basque Autonomous Community. In addition, those responsible for the sports clubs were contacted by telephone, and the teams were visited during their training sessions to increase the number of responses. Participation in the study was voluntary, and the data were anonymous and confidential. The study was approved by the ethical committee of the authors’ institution (Ref: ETK-30/22-23), and all participants signed an informed consent form. The participating athletes had the option to request the results of the study.

### 2.4. Statistical Analysis

Initially, a descriptive study of the sample was conducted with sociodemographic data and the variables of sports practice presented through the number of responses (N) and percentages (%). To describe the variables statistically, the arithmetic mean (M), standard deviation (SD) and the minimum and maximum values were calculated. Reliability analysis was performed by calculating Cronbach’s coefficients, establishing a minimum criterion of 0.70 for the total scales. To compare whether there were differences between the variables, we proceeded with parametric inferential statistics, specifically the *t*-test for independent groups, and ANOVA to compare the independent means in more than two groups. For variables that did not follow a normal distribution, the Mann—Whitney U test was used to compare two independent samples and the Kruskal—Wallis test for more than two. The approximation to normal distribution was tested using the Kolmogorov—Smirnoff test for groups larger than 50 subjects and the Shapiro—Wilk test for smaller groups. The effect size was calculated using Cohen’s d test. Cohen’s d values of <0.20, 0.20–0.50, 0.51–0.80 and >0.80 were rated as trivial, small, moderate and large effects, respectively [47]. Finally, we analysed the Pearson correlation coefficients (r) to examine the relationships between the variables. The analyses were conducted using SPSS (Statistical Package for Social Sciences), version 28.0. The confidence level established was *p* ≤ 0.5.

## 3. Results

The scales indicated good internal consistency, as shown in Table 2. Once the reliability of the scales had been analysed, their discriminant validity was analysed in the sample of athletes with physical disabilities. Discriminant validity was analysed by means of the correlation of the construct with its indicators, whereby the square root of the average variance extract (AVE) must be higher than the correlation between constructs [48], as shown in Table 3.

### Influence of the Variables on Psychological Capital and Life Satisfaction

In accordance with the research objective, the levels of life satisfaction and psychological capital of physically disabled athletes are described according to their age, gender, origin of disability, degree of dependence and level of competition.

Age did not contribute statistically significant differences in psychological capital (F(79) = 1.09, *p* = 0.35) and life satisfaction (χ^2^(79) = 5.73, *p* = 0.12). 

Gender showed significant differences in total psychological capital (t(79) = 2.36, *p* = 0.02), being higher among men (M = 4.51; SD = 0.62) than in women (M = 4.01; SD = 0.79). In addition, statistically significant differences were found in two of the four dimensions of psychological capital. The self-efficacy dimension (t(79) = 2.42, *p* = 0.02) was higher among men (M = 4.63; SD = 0.76) than among women (M = 4.03; SD = 0.83). Differences were also found in the hope dimension (t(79) = 2.18, *p* = 0.03), where men presented higher scores (M = 4.50; SD = 0.73) than women (M = 3.95; SD = 0.96). In the life satisfaction dimension (t(79) = 1.72, *p* = 0.09), men (M = 26.37; SD = 5.30) also reported higher scores than women (M = 23.18; SD = 7.77) and, although the difference is not statistically significant, the effect size shows a moderate difference (d = 0.48).The complete results are shown in Table 4.

Regarding the degree of dependence, results close to statistical significance were obtained in psychological capital, specifically in the dimensions of hope (F(79) = 1.78; *p* = 0.06) and life satisfaction (χ^2^ = 4.79; *p* = 0.09). In the dimension of hope, the athletes with Grade 0 (M = 4.64; SD = 0.77) indicated a higher degree of hope than those with Grade I (M = 4.20; SD = 0.60) and Grades II and III (M = 4.27; SD = 0.90). Notably, in terms of the level of life satisfaction, athletes with Grade 0 (M = 27.44; SD = 4.90) showed higher values than those with Grades II and III (M = 23.36; SD = 7.35). Table 5 presents the results. 

Regarding the origin of the disability, no statistically significant results were found in psychological capital (t(79) = −0.60, *p* = 0.55) and life satisfaction (U(79) = 1.95, *p* = 0.16). Table 6 presents the results.

Regarding the level of competition, the psychological capital in the dimension of self-efficacy (F = 3.43; *p* = 0.03) showed statistically significant results in which international athletes reported a higher level of self-efficacy (M = 4.79; SD = 0.74) than regional athletes (M = 4.24; SD = 0.76). Table 7 presents the results.

Finally, stepwise hierarchical regression analysis was performed to determine the predictive capacity of the variables analysed under life satisfaction. The results (Table 8) show that the PsyCap predictors were able to explain 17% of life satisfaction, followed by the athletes’ degree of dependence, which explained an additional 5%. The variables of age, sex, origin of disability and level of competition did not prove significant.

## 4. Discussion

This study analysed the levels of life satisfaction and psychological capital in a sample of physically disabled athletes according to several variables, namely, age, gender, degree of dependence, type of disability and level of competition. In this section, we will compare the results obtained in the present study with those obtained in related studies.

### 4.1. Sociodemographic Variables

With respect to H1, no significant differences were found in relation to age, in accordance with previous studies. For example, Tejero-González et al. [30] analysed life satisfaction and self-efficacy (the dimension of psychological capital) among wheelchair-playing athletes and did not obtain significant results. This may be due to the homogeneity of the sample, composed only of older athletes.

In terms of H2, the results generally did not show statistically significant results regarding to gender. First, results were obtained in relation to psychological capital and in the dimensions of self-efficacy and hope. Several studies confirm this result: men have a greater tendency towards well-being linked to autonomy, whereas women experience well-being related to affective and emotional aspects [49,50]. As for life satisfaction, men showed higher values of life satisfaction than women. Notably, in previous research, women with disabilities showed higher values of life satisfaction than men [51]. This result may be attributed to the reduced female participation in our study and the lack of variables, such as frequency or type of sport. Given the disparity in the distribution of participants by gender, this result could be biased. It is suggested that future research take this result into account and ensure more equitable participation.

### 4.2. Disability Variables

Regarding to H3, despite the degree of dependence did not reach statistical significance, it was obtained a sample size close to where athletes with a lower degree of dependence indicated higher levels of life satisfaction and psychological capital, particularly in the dimension of hope. Our result is confirmed by Lucas-Castro and Salvador-Carulla [34], who concluded that people with lower degrees of dependence showed higher levels of life satisfaction. In addition, engaging in sports increases life satisfaction and quality of life in people with physical disabilities, which is also reported by Yazicioglu et al. [35]. Thus, in our study, a positive correlation was observed between the athletes’ degree of dependence and their general health, autonomy and levels of life satisfaction and hope.

With respect to H4, no statistically significant results were found in relation to the origin of the disability and the psychosocial variables analysed. However, it should be noted that athletes with an innate disability rate life satisfaction higher than those with an acquired disability. Nevertheless, authors such as Hopper [37] pointed out that people with innate disabilities are at greater risk of experiencing low levels of self-esteem. For this reason, sports can be a powerful tool for athletes with innate disabilities, as sports can help improve their well-being and life satisfaction, as observed in the results. 

Regarding psychological capital, athletes with acquired disabilities showed higher values in terms of resilience. This is in accordance with recent studies stating that people with acquired disabilities have greater well-being and psychological capacities than those with innate disabilities, which can be attributed to their ability to adapt to new conditions and face new adversities [39]. In summary, athletes with innate disabilities showed greater life satisfaction, while those with acquired disabilities showed a greater ability to cope and adapt to adverse situations.

### 4.3. Variables Related to Sports Practice

The present study has also shown relevant data regarding the origin of the disability and the level of competition (Table 1): the majority of international athletes have a supervening disability (n = 23) compared to a small number of athletes with an innate disability (n = 8). This phenomenon may be attributed to the absence of acquired sports habits among people with innate disabilities due to personal and environmental barriers, such as a lack of opportunities, as well as material and human resources [6,7,52,53]. Athletes with acquired disabilities have experienced early participation in sports activities and have greater experiences and acquired sports habits, thus facilitating greater adherence to sports practice after acquiring their disabilities.

Regarding H5, that is, considering athletes’ levels of competition, this study obtained statistically significant results, wherein athletes with higher levels of competition have greater life satisfaction and psychological capital. Studies by Horvat et al. [42] and Wisniowska et al. [43] confirmed our results; that is, athletes with physical disabilities who compete at the international level have higher levels of life satisfaction than others who compete at national or regional levels. Furthermore, athletes competing at the international level have higher levels of dedication in terms of frequency and demand for training than those competing at lower levels. As a result, a positive relationship is established between sports frequency and dedication to sports on the one hand and psychological capital and life satisfaction on the other.

In relation to the instruments used, it is worth mentioning that the Psycap variables have shown a high correlation with each other. This is due to the fact that the conjunction of the four variables constitutes the Psycap construct proposed by Luthans et al. [25]. It should be noted that the results obtained show a high similarity with the data shown in the study of the adaptation of the questionnaire to the sporting environment [24]. Following the recommendations of previous studies, the measurement of all the variables as a whole provides relevant information in the study of strengths and well-being [23,24].

Finally, our study confirmed the notion that psychological capital is a predictor of life satisfaction, followed by the degree of dependence. At the scientific level, these findings validate the results obtained in previous research [27,34] and support the importance of developing specific programmes aimed at improving psychological capital in athletes with physical disabilities due to their fundamental role in improving life satisfaction. In the design of these programmes, it was also considered essential to assess the athletes’ degree of dependence, as this factor exerted a significant influence. In summary, the focus was on the optimal development of their psychological capabilities while improving their well-being. This makes it possible to achieve an optimal state of well-being among physically disabled athletes.

## 5. Conclusions

The present study is not without limitations. First, it is aimed at a specific population, which means it is not probabilistic or random. Thus, the results cannot be generalised to all athletes with physical disabilities. To date, no research has analysed psychological capital in athletes with physical disabilities. This research provides relevant data on the psychological strengths of this population in this sample. Regarding the variables analysed, our results show that gender, degree of dependence and level of competition influence the psychological capital and life satisfaction of athletes. This information can be useful for coaches or sports psychologists, who can guide their athletes to improve their psychological strengths and well-being, which are essential in avoiding abandonment, especially in women; among them we would highlight the importance of working on the athlete’s self-efficacy, autonomy and hope future lines of research should include larger and more representative samples of the group, as well as explore more psychosocial (e.g., psychological well-being or perceived social support) and sporting (e.g., type of sport practiced, frequency of sport practice and level of experience) variables.

## Figures and Tables

**Table 1:** Personal characteristics of the participating athletes.

Competition Level	Middle Age (M = 39.94)	Gender		Origin of Disability	
		Male (N = 68)	Female (N = 11)	Innate (N = 29)	Acquired (N = 50)
International	39.26	28	3	8	23
National	39.12	24	1	12	13
Regional	41.74	16	7	9	14

**Table 2:** Reliability analysis of the measurement tools.

Measuring Tool		Alpha
Psychological Capital (PsyCap)	Self-efficacy	0.79
	Hope	0.80
	Resilience	0.79
	Optimism	0.67
	Total	0.89
Satisfaction with Life Scale (SWLS)		0.84

**Table 3:** Descriptive statistics, correlations and discriminant validity of the Sport Psychological Capital Questionnaire (SPCQ).

	M (SD)	0	1	2	3	4
0. PsyCap	4.44 (0.65)	1				
1. Self-efficacy	4.55 (0.79)	0.845	0.647			
2. Hope	4.42 (0.78)	0.829	0.509	0.707		
3. Resilience	4.44 (0.84)	0.797	0.591	0.509	0.707	
4. Optimism	4.15 (0.93)	0.751	0.538	0.592	0.534	0.720

**Table 4:** Psychosocial variables as a function of the genre. Student’s *t*-test for independent samples.

		Genre						
		Male (N = 68)		Female (N = 11)				
		M	(SD)	M	(SD)	Statistics	*p*	d
Psychological Capital	Self-efficacy	4.63	0.76	4.03	0.83	t = 2.42	0.02 **	0.76
	Hope	4.50	0.73	3.95	0.96	t = 2.1	0.03 **	0.76
	Resilience	4.50	0.79	4.09	1.07	U = 29	0.28	0.12
	Optimism	4.18	0.91	3.95	1.08	U = 31	0.43	0.09
	Total	4.51	0.62	4.01	0.79	t = 2.36	0.02 **	0.64
Life Satisfaction		26.37	5.30	23.18	7.77	t = 1.72	0.09	0.48

M: mean; SD: standard deviation. t: student’s *t*-test; U: Mann–Whitney U test. ** *p* ≤ 0.05.

**Table 5:** Psychosocial variables as a function of the degree of dependency. Student’s *t*-test for independent samples.

		Degree of Dependency								
		Grade 0 (N = 36)		Grade I (N = 21)		Grade II or III (N = 22)				
		M	(SD)	M	(SD)	M	(SD)	Statistics	*p*	d
Psychological Capital	Self-efficacy	4.58	0.78	4.45	0.80	4.59	0.83	F = 0.21	0.81	0.00
	Hope	4.64	0.77	4.20	0.60	4.27	0.90	F = 2.81	0.06	0.06
	Resilience	4.57	0.76	4.18	0.81	4.48	0.97	χ^2^ = 2.84	0.24	0.04
	Optimism	4.19	1.02	4.24	0.70	4.00	1.00	χ^2^ = 0.80	0.66	0.01
	Total	4.56	0.62	4.28	0.64	4.39	0.75	F = 1.18	0.31	0.03
Life Satisfaction		27.44	4.90	26.00	4.35	23.36	7.35	χ^2^ = 4.79	0.09	0.06

M: mean; SD: standard deviation. χ^2^: Kruskal–Wallis; F: Analysis of variance (ANOVA).

**Table 6:** Psychosocial variables as a function of the origin of disability. Student’s *t*-test and Mann–Whitney U test for independent samples.

		Origin of Disability						
		Innate (N = 29)		Acquired (N = 50)				
		M	(SD)	M	(SD)	Statistics	*p*	d
Psychological Capital	Self-efficacy	4.53	0.87	4.56	0.75	U = 0.01	0.90	0.01
	Hope	4.34	0.91	4.47	0.71	t = −0.66	0.51	0.16
	Resilience	4.35	0.89	4.49	0.81	U = 0.80	0.37	0.06
	Optimism	4.03	0.97	4.22	0.92	U = 0.34	0.56	0.07
	Total	4.37	0.80	4.48	0.58	t = −0.60	0.55	0.16
Life Satisfaction		26.79	6.02	25.42	5.59	U = 1.95	0.16	0.23

M: mean; SD: standard deviation. t: student’s *t*-test; U: Mann—Whitney U test.

**Table 7:** Psychosocial variables as a function of the competition level. Student’s *t*-test for independent samples.

		Competition Level								
		Regional (N = 23)		National (N = 25)		International (N = 31)				
		M	(SD)	M	(SD)	M	(SD)	Statistics	*p*	d
Psychological Capital	Self-efficacy	4.24	0.76	4.53	0.80	4.79	0.74	F = 3.43	0.03 **	0.08
	Hope	4.11	0.91	4.49	0.73	4.60	0.68	χ^2^ = 3.44	0.18	0.04
	Resilience	4.43	0.94	4.26	0.81	4.59	0.79	χ^2^ = 1.92	0.38	0.02
	Optimism	4.07	0.96	4.04	1.00	4.31	0.87	χ^2^ = 0.78	0.67	0.01
	Total	4.22	0.70	4.40	0.68	4.63	0.59	F = 2.65	0.07	0.06
Life Satisfaction		23.91	7.12	25.88	5.46	27.45	4.42	F = 2.60	0.08	0.06

M: mean; SD: standard deviation. χ^2^: Kruskal–Wallis test; F: Analysis of variance (ANOVA). ** *p* ≤ 0.05.

**Table 8:** Descriptive statistics, correlations and discriminant validity of the Psychological Capital Scale.

	Life Satisfaction			
Predictor	B	Error	Beta	*p*
Step 1				
PsyCap	3.57	0.89	0.41	<0.001
R^2^ = 0.17 R^2^ adjusted = 0.16 F = 15.90 *p* < 0.001				
Step 2				
PsyCap	3.35	0.87	0.38	<0.001
Degree of dependence	−1.29	0.57	−0.23	<0.001
R^2^ = 0.22 R^2^ adjusted = 0.20 F = 10.93 *p* < 0.001				

Excluded variables: age, sex, origin of disability and level of competition.

## Data Availability

The data that support the findings of this study are available from the corresponding author upon reasonable request.
